# Correlation of BUB1 and BUB1B with the development and prognosis of endometrial cancer

**DOI:** 10.1038/s41598-024-67528-2

**Published:** 2024-07-24

**Authors:** Huicong Zhang, yuhao li, Huixia Lu

**Affiliations:** 1 Clinical Medicinal College of Dali University, Dali City, 671000 Yunnan Province China; 2 Yunnan Provincial Key Laboratory of Entomological Biopharmaceutical R&D, Dali University, Dali, 671000 Yunnan Province China; 3 West China School of Basic Medical Sciences and Forensic Medicine,, Sichuan University, 610041 Chengdu, China

**Keywords:** BUB1, BUB1B, Endometrial cancer, Development, Prognosis, Clinicopathological characteristics, Cancer, Computational biology and bioinformatics

## Abstract

This study aimed to evaluate the expression and clinical significance of budding uninhibited by benzimidazole 1 (BUB1) and BUB1 mitotic checkpoint serine/threonine kinase B (BUB1B) in endometrial carcinoma (EC). BUB1 and BUBIB expressions were evaluated by bioinformatics. Protein expression, clinical features, prognosis and immune cell infiltration were explored in 20 EC tumors. siRNA was used to evaluate BUB1 and BUBIB function in EC cells. BUB1 and BUBIB were highly expressed in 26 cancers. BUB1 was associated with overall survival (OS) in eight cancers and disease-free survival in ten; BUB1B was associated with OS in nine cancers and DFS in eleven. BUB1 and BUBIB exhibited high frequencies of gene changes (mainly mutations, > 5%) in cancer. BUB1 was negatively correlated and BUB1B was positively correlated with cancer-associated fibroblasts and endothelial cell infiltration. BUB1 and BUBIB knockdown decreased migration and invasion in EC cells. High BUB1 expression correlated with tumor malignant phenotypes (*P* < 0.05). High BUB1 mRNA expression reduced OS (*P* = 0.00036) and recurrence-free survival (*P* = 0.0011). High BUB1B mRNA expression reduced OS (*P* = 0.0024). BUB1/BUB1B correlated with activated CD8 + T and CD4 + T cell infiltration. BUB1 and BUBIB are highly expressed and correlated with clinicopathological characteristics in EC. BUB1 and BUBIB are potential prognosis markers and immunotherapy targets.

## Introduction

Endometrial carcinoma (EC) accounts for 20–30% of malignancies of the female reproductive tract^1,2^. In recent years, the incidence of EC has been increasing^3^. Over 400,000 new EC cases and 90,000 EC-related deaths were reported worldwide in 2020; Every year around 65,000 females develop uterine cancer in the USA alone, > 90% of these cases are of endometrial origin^4,5^. Even after treatment including surgery, platinum-based chemotherapy and radiotherapy, 30% of advanced EC cases have a poor prognosis^6–8^. Therefore, timely diagnosis and treatment are required to improve the prognosis of EC patients.

Cancer cells are characterized by uncontrolled proliferation and aneuploidy that is associated with abnormal cell division processes^9^. The spindle assembly checkpoint (SAC) is an important monitor during cell division that prevents aneuploidy and maintains genomic stability by monitoring the kinetochore-microtubule attachment and ensures correct division^10–12^. Budding uninhibited by benzimidazole 1 (BUB1) and its paralogous homolog BUB1 mitotic checkpoint serine/threonine kinase B (BUB1B) are members of the SAC protein family. The physical interaction mediated by a conserved N-terminal region between BUB1 and BUB1B with blinkin protein^13^ leads to the cooperation of BUB1 with BUB1B in the mitotic checkpoint kinetochore localization^14^. BUB1 and BUB1b exhibit critical biological functions by preventing premature mitotic chromosome segregation and reducing aneuploidy^15^. Mutations in BUB1 and BUB1B genes have been identified in cancer. High expression of BUB1 promotes the proliferation and invasion of gastric cancer cells through the Wnt/β-catenin signaling pathway, and down-regulation of BUB1 induces S-phase arrest of liver cancer cells^16,17^. Overexpression of BUB1B can regulate the glycolysis of lung adenocarcinoma cells and bind to zinc finger protein 143 (ZNF143) to promote proliferation, migration and invasion. Highly expressed BUB1B also promotes multiple myeloma cell proliferation through the CDC20/CCNB axis^18,19^. Both BUB1 and BUB1B have been associated with the high proliferative activity of tumor cells and adverse clinical outcomes of various solid tumors^20^. BUB1 and BUB1B genes were identified as significant hub differentially expressed genes in epithelial ovarian cancer associated with a poor prognosis^21^. Previous studies have revealed an upregulation of BUB1 and BUB1B in EC^22^. However, the expression and roles of BUB1 and BUB1B in pan-cancer and EC is unknown.

In this study, we examined the expression and clinical significance of using experimental and bioinformatics methods to explore the possibility of BUB1 and BUB1B as biomarkers and diagnosis and therapeutic targets in EC.

## Methods

### Tissue samples

This study included endometrial and paracancerous tissue samples from 20 EC patients who underwent surgical treatment in the Gynecology Department of the First Affiliated Hospital of Dali University from June 2022 to October 2022. The Ethical Committee of the Hospital for Clinical Research approved this study, and all samples were obtained with the patients’ informed consent. All methods were performed in accordance with the Declaration of Helsinki (DFY20220817001). Inclusion criteria were as follows: primary endometrioid carcinoma confirmed by postoperative pathology and no preoperative neoadjuvant radiotherapy and endocrine therapy. Exclusion criteria were as follows: previous or current combination of other tumors and the combination of severe medical or surgical disease or other contraindications.

### Immunohistochemical (IHC) staining

Endometrial and paracancerous tissues were fixed with formalin and embedded in kerosene. Tissue sections (4 µm thick) were deparaffinized and subjected to antigen retrieval, followed by treatment with 3% hydrogen peroxide and serum treatment for 30 min. Samples were incubated with primary antibody (rabbit anti-human BUB1 and BUBIB monoclonal antibody, 1:200) (Sanying Biotechnology Co. Ltd., Wuhan, Hubei, China), followed by incubation with secondary antibody (HRP-labeled goat anti-rabbit II antibody, 1:200) (Zhongshan Jinqiao Biotechnology Co. Ltd., Beijing, China). The grade and percentage of IHC-stained cells were evaluated using a light microscope (× 400) in five separate microscopic fields^23^.

### Bioinformatics analysis

#### Gene expression analysis

We used the TIMER 2.0 database (http://timer.cistrome.org/) to obtain BUB1 and BUB1B gene expressions in different tumors in TCGA. We also performed analysis of TCGA clinical data combined with GTEx using GEPIA 2 (http://gepia2.cancer-pku.cn/). Data were analyzed using |log2fold change|> 1 and *P* < 0.01. BUB1 and BUB1B expression in normal endometrial and EC tissues was derived using the XianTao database, and the ROC curve was plotted.

#### Survival analysis

We used the GEPIA2 database to obtain the relationship between BUB1 and BUB1B mRNA expression levels and overall survival (OS) and disease-free survival (DFS) of all tumors in TCGA. Kaplan–Meier Plotter database (http://kmplot.com/analysis/) was used to explore the prognostic significance of BUB1 and BUBIB, including overall survival and recurrence-free survival (RFS), in EC.

#### Genetic variation analysis

cBioPortal database (http://cbioportal.org/) was used to examine variant frequency, mutation type, and copy number variations (CNVs). The genetic variants of BUB1 and BUB1B in EC were also obtained.

#### Immune infiltration analysis

TIMER 2.0 database was used to analyze the relationship between BUB1 and BUB1B expression and immune cell infiltration, including cancer-associated fibroblasts, neutrophils and endothelial cells, in all tumors. We used the TISIDB database to obtain the relationship between BUB1 and BUB1B expression and immune cell infiltration and immunomodulators in EC tissues.

#### Clinicopathological analysis

The ULCAN database was used to obtain the expression of BUB1 and BUB1B mRNA in EC tissues in different stages, menopausal status, histological subtypes and TP53 mutation status.

#### Enrichment-related genes

The STRING database (https://string-db.org) was used to analyze the BUB1- and BUB1B-related protein interaction networks. The top 100 related genes were obtained using the “Similar Genes Detection” module in GEPIA2. Pearson analysis was performed between BUB1 and BUBIB in various tumors and the top five significantly related genes with the strongest correlation were selected for paired gene–gene Pearson correlation analysis. The 100 tumor-related genes obtained from GEPIA2 were subjected to functional clustering analysis using “R” software.

### Cell culture

Human endometrial cancer Ishikawa cells were cultured in DMEM (with 10% fetal bovine serum and 1% penicillin/streptomycin) in an incubator at 37 ℃, 5% CO_2_.

#### Cell transfection

Transfection with siRNABUB1 and siRNA-BUB1B (Jima Pharmaceutical Co., Ltd., Shanghai, China) was performed using a Lipofectamine™ 2000 kit following the manufacturer’s instructions. The cell culture medium was changed after 6 h.

#### CCK8 assay

Cells in 96-well plates were transfected as indicated. After 24 h, 48 h and 72 h, 100 µl CCK8 reagent and cell culture medium (1:9) were added. Cells were incubated for 1 h and the absorbance of each well at 450 nm was measured.

#### Wound healing assay

Cells in 6-well plates were transfected as indicated. At 24, the cell monolayer was scratched with a 10 µl pipette tip. The cell scratch was photographed under a microscope at 0 h and 24 h. The wound healing rate (%) was determined as = (d_0 h_–d_24 h_)/d_0 h_ × 100%, where d_0h_ = wound width at 0 h and d_24h_ = wound width at 24 h.

#### Transwell assays

Cells were inoculated into the top chamber of a Transwell containing serum-free DMEM pre-layered with Matrigel. The bottom chamber contained 500 µl DMEM high sugar medium and 20% fetal bovine serum. After the indicated times, cells were fixed in 4% paraformaldehyde, stained with 0.1% crystal violet for 10 min and photographed.

### qPCR

Total cellular RNA was extracted using the Centrifugal Column Total RNA Extraction Kit (Nuoweizan Biotechnology Co., Ltd., Nanjing, Jiangsu, China). cDNA was synthesized using the PrimeScript™ RT Kit (Takara Bio Inc., Tokyo, Japan), and RT-qPCR was performed using the PowerUp SYBR Master Mix (Thermo Fisher Scientific Inc., Waltham, MA, USA). Primers are listed in Table S1. The cycling conditions were as follows: pre-denaturation at 95 ℃ for 30 s, followed by 40 cycles at 95 ℃ for 5 s and 60 ℃ of annealing for 10 s. Melting curve acquisition was performed at 95 ℃ for 15 s, 60 ℃ for 60 s and 95 ℃ for 15 s, and primer specificity was determined. GADPH mRNA was used as an internal control for normalization, and gene expression was normalized by the 2^−ΔΔCt^ method.

### Statistical methods

All experiments were repeated three times. Data were analyzed using SPSS 25.0 statistical software. LSD t-test was performed for comparison between two groups; ANOVA was used for comparison between multiple groups. Data are expressed as mean ± standard deviation (x ± s); Pearson correlation was used for correlation study between measurement data. *P* < 0.05 indicated statistical significance.

## Results

### BUB1 and BUBIB are differentially expressed in pan-cancer

**Figure 1:**
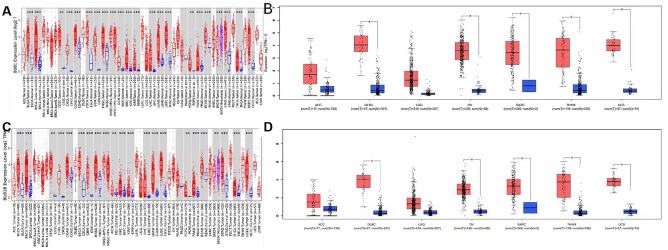
Analysis of BUB1 and BUB1B gene differential expression in tumors. (**A**) Analysis of BUB1 gene expression in tumors in TIMER 2.0 database; (**B**) Analysis of BUB1 gene expression analysis in tumors in GEPIA2 database; (**C**) Analysis of BUB1B gene expression analysis in tumors in TIMER 2.0 database; (**D**) Analysis of BUB1B gene expression analysis in tumors in GEPIA2 database. *P < 0.05.

Compared with BUB1 gene expression in normal tissues, BUB1 gene was expressed at higher levels in BLCA (bladder urothelial carcinoma), BRCA (breast invasive carcinoma), CHOL (cholangiocarcinoma), COAD (colon adenocarcinoma), ESCA (esophageal carcinoma), GBM (glioblastoma multiforme), HNSC (head and neck squamous cell carcinoma), KICH (kidney chromophobe), KIRC (kidney renal clear cell carcinoma), KIRP (kidney renal papillary cell carcinoma), LIHC (liver hepatocellular carcinoma), LUAD (lung adenocarcinoma), LUSC (lung squamous cell carcinoma), PRAD (prostate adenocarcinoma), READ (rectum adenocarcinoma), SKCM (skin cutaneous melanoma), STAD (stomach adenocarcinoma), TGCT (testicular germ cell tumor), UCEC (uterine corpus endometrial carcinoma) (*P* < 0.001), CESE (cervical squamous cell carcinoma and endocervical adenocarcinoma), and PCPG (pheochromocytoma and paraganglioma) (*P* < 0.01). Only PAAD (pancreatic adenocarcinoma) showed no difference in BUB1 gene expression (Fig. 1A). BUB1B gene was highly expressed in BLCA, BRCA, CHOL, COAD, ESCA, GBM, HNSC, KIRC, KIRP, LIHC, LUAD, LUSC, PRAD, READ, STAD, THCA, UCEC (*P* < 0.001), KICH, CESC, PCPG, and SKCM (*P* < 0.01); only PAAD showed no difference in BUB1 gene expression (Fig. 1B).

BUB1 was highly expressed in DLBC (lymphoid neoplasm diffuse large B-cell lymphoma), OV (ovarian cancer), SARC (sarcoma), THYM (thymoma) and USC (uterine carcinosarcoma) compared with normal tissues; no difference was observed in ACC (adrenocortical carcinoma) and LGG (brain lower grade glioma) (Fig. 1C). BUB1B was highly expressed in DLBC, OV, SARC, THYM and USC (*P* < 0.05); no difference was observed in ACC and LGG (Fig. 1D).

### BUB1 and BUBIB are highly expressed in EC

**Figure 2:**
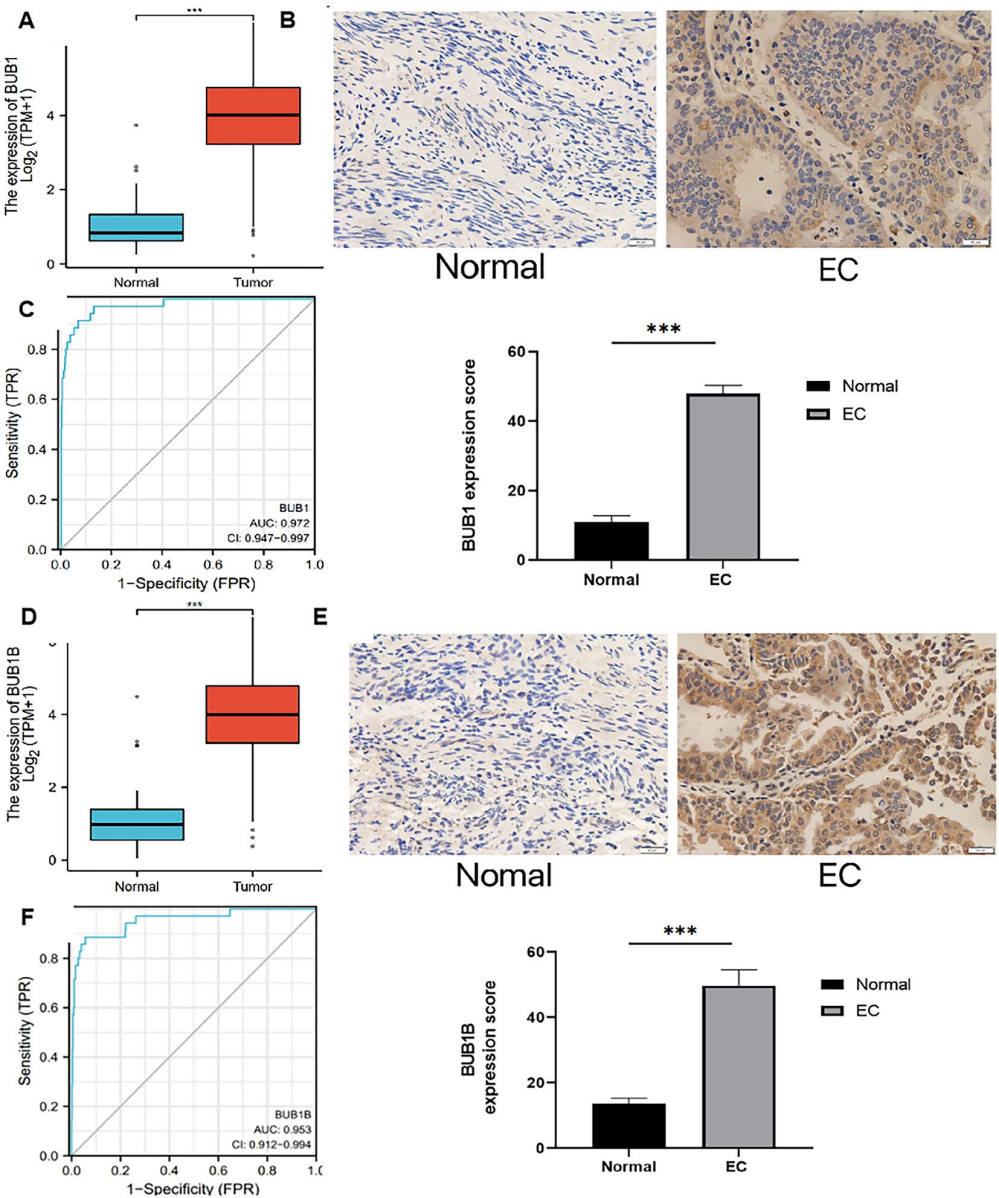
BUB1 and BUB1B expression in EC. (**A**) BUB1 expression in normal endometrium and EC (XianTao database); (**B**) BUB1 expression in normal endometrium and EC (IHC); (**C**) BUB1B expression in normal endometrium and EC (XiaoTao database); (**D**) BUB1B expression in normal endometrium and EC (IHC); (**E**) BUB1 ROC curve in EC; (**F**) BUB1B ROC curve in EC.

BUB1 and BUB1B mRNAs were upregulated in EC tissues, as determined by XiaoTao tool analysis (Fig. 2A,C). IHC showed that BUB1 and BUBIB were positively expressed in EC tissues (*P* < 0.05) and barely expressed in paracancerous tissues, consistent with the bioinformatics analysis (Fig. 2B,D). BUB1 and BUB1B proteins were mainly located in the cytoplasm.

ROC curve showed that the high expressions of BUB1 (AUC = 0.972, CI 0.947–0.997) and BUB1B (AUC = 0.953, CI 0.912–0.994) had high accuracy in the prediction of EC (Fig. 2E,F).

### High expression of BUB1 and BUBIB leads to poor prognosis in pan-cancer

GEPIA2 analysis indicated that high BUB1 expression was associated with OS in ACC, KIRC, KIRP, LGG, LIHC, LUAD, LUSC, and PAAD (*P* < 0.05) (Fig. S1A) and associated with DFS in ACC, KIRC, KIRP, LGG, LIHC, MESO, PAAD PRAD, SARC and THCA (*P* < 0.05) (Fig. S1B). High BUB1B expression was associated with OS in ACC, KIRC, KIRP, LGG, LIHC, LUAD, MESO, PAAD, and SARC (*P* < 0.05) (Fig. S1C) and associated with DFS in ACC, CHOL, KIRC, KIRP, LGG, LIHC, LUAD, PAAD, PRAD, SARC and THCA (*P* < 0.05) (Fig. S1D).

### High expression of BUB1 and BUBIB leads to poor prognosis in EC

High BUB1 mRNA expression was associated with poor OS (*P* = 0.00036) and RFS (*P* = 0.0011) in EC. High BUB1B mRNA was associated with poor OS (*P* = 0.0024) but had no effect on RFS (*P* = 0.064) in EC (Fig. S2A,B).

### BUB1 and BUBIB variation tumors frequently show other genetic variations in pan-cancer

The frequency of BUB1 gene alterations in melanoma, bladder cancer, and EC was high (frequency > 5%) and the gene alterations mainly included mutations (Fig. S3A), including missense mutations, amplifications and profound deletions. The most common CNVs were diploidy, gain and shallow deletion (Fig. S3B). ARAP-AS1, TTN, MUC16, LRP1B, FLG, CSMD3, TP53, SLTL2-IT1, MT1F, and PTPRQ genetic alterations were more common in the BUB1 variant group (Fig. S3C).

BUB1B gene alterations were more frequent in EC and melanoma (frequency > 5%) and the alterations were predominantly mutations (Fig. S3D). All PM (pleural mesothelioma) (frequency > 4%) had BUB1B gene deep deletion. Missense mutations, deep deletions and amplification were the main types of variations. The most common CNVs were diploidy, gain and shallow deletion (Fig. S3E). KIZ-ASL, LNCNEF, linc01721, linc00261 LINC00656, LINC01727, LINC01427, LINC01432, LINC01431, and CST13P were common in the BUB1B gene alteration group (Fig. S3F).

### BUB1 and BUBIB genetic variation had no effect on survival of EC patients

**Figure 3:**
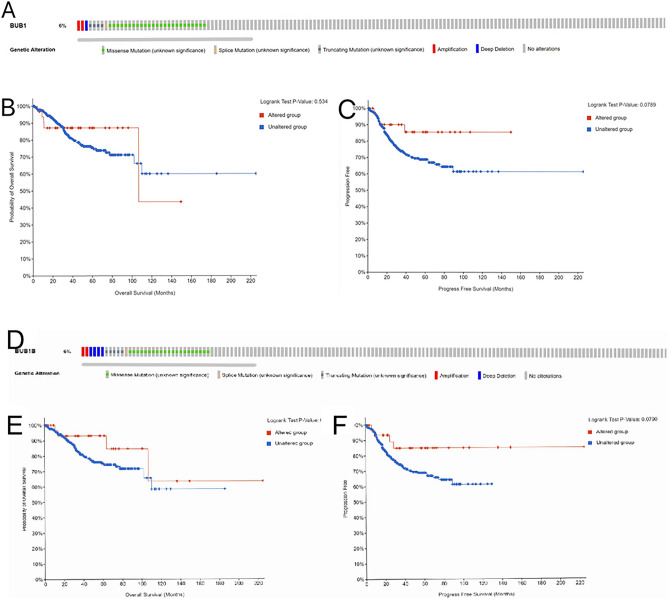
BUB1 and BUB1B gene variations in EC. (**A**) BUB1 gene alteration in EC; (**B**) BUB1 gene alteration in relation to OS in EC; (**C**) BUB1 gene alteration in relation to PFS in EC. (**D**) BUB1B gene alteration in EC; € BUB1B gene alteration in relation to OS in EC; F. BUB1B gene alteration in relation to PFS in EC.

BUB1 gene alteration occurred in 6% (33/509) of EC patients and the main type was missense mutation (Fig. 3A). The effect on OS (*P* = 0.534) (Fig. 3B) and PFS was not significant (*P* = 0.0789) (Fig. 3C). BUB1B gene alteration occurred in 6% (33/509) of EC patients, and the main type was missense mutation (Fig. 3D); it did not have a significant effect on OS (*P* = 0.219) (Fig. 3E) and PFS (*P* = 0.0790) (Fig. 3F).

### BUB1 and BUBIB were associated with multiple immune infiltrations in pan-cancer

We used TIMER, CBERSORT, TIDE, XCELL, MCPCOUNTER, and QUANTISEQEPIC databases to explore the correlation between BUB1and BUB1B and cancer-associated fibroblast (CAF), endothelial cell and neutrophil infiltration levels in different tumors in TCGA database. BUB1 expression level was negatively correlated with CAF infiltration of BRCA and TGCT and endothelial cell infiltration of BRCA, KIRC, LUAD, STAD, THCA, and THYM and positively correlated with neutrophil infiltration of COAD and endothelial cell infiltration of KIRP and LGG (Fig. S4A–C). BUB1B expression level was negatively correlated with the CAF infiltration of BRCA, HNSC-HPV + (HPV-associated head and neck squamous cell carcinoma), TGCT, and THYM and endothelium infiltration of BRCA, KIRC, LUAD, STAD, and THYM (Fig. S4D–F).

### BUB1 and BUBIB was associated with immune infiltration in EC

**Figure 4:**
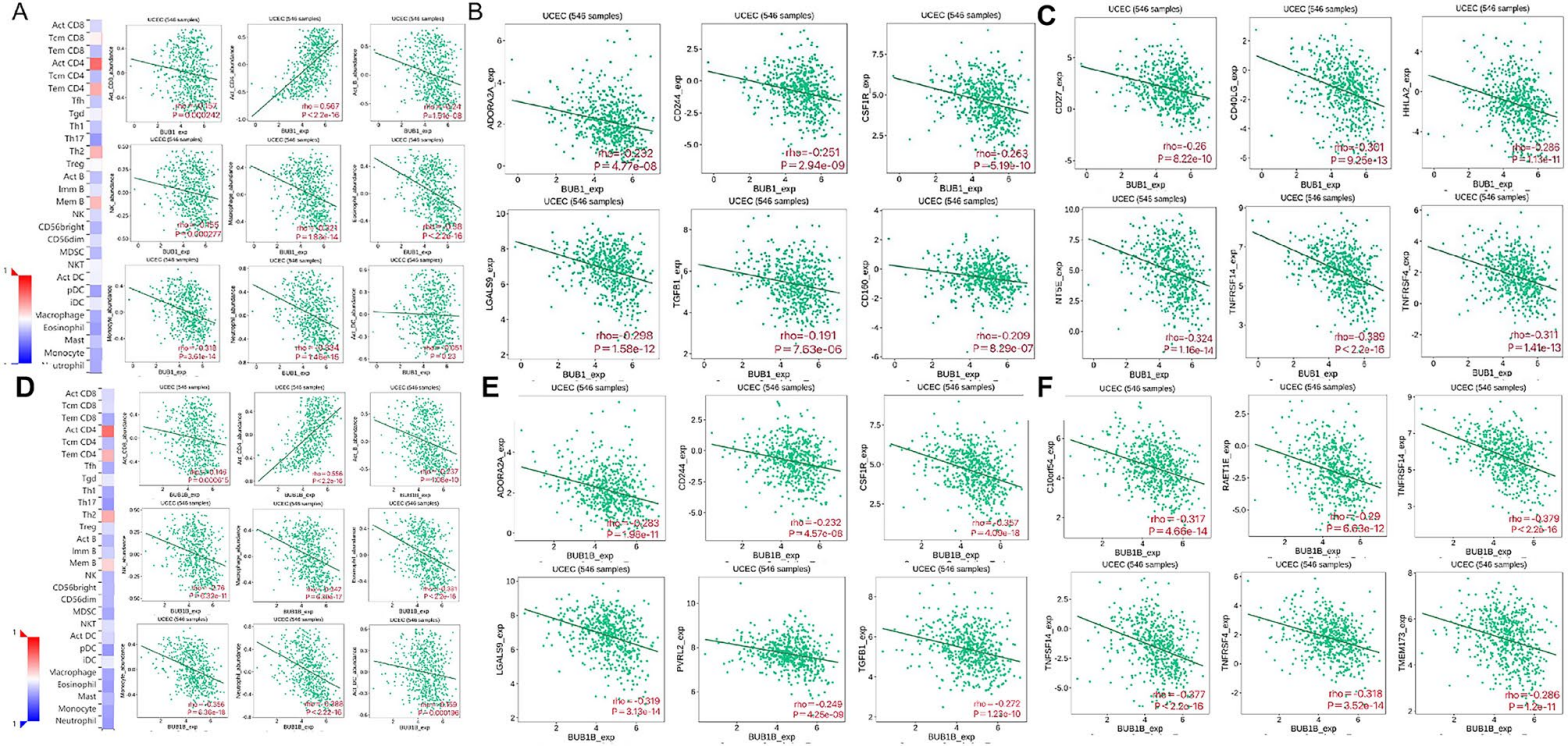
BUB1 or BUB1B and EC immune filtration. (**A**) BUB1 expression was associated with TIL abundance in EC; (**B**) BUB1 expression was associated with immunosuppressants in EC; (**C**) BUB1 expression and immunostimulants in EC. (**D**) BUB1B expression was associated with TIL abundance in EC; E. BUB1B expression was associated with immunosuppressants in EC; (**F**) BUB1B expression and immunostimulants in EC.

BUB1 was associated with abundance of a variety of tumor-infiltrating lymphocytes (TILs) and mostly showed a negative correlation (Fig. 4A). BUB1 level was associated with act-CD8 + T cells (rho = − 0.157), act-CD4 + T cells (rho = 0.5676, act-B cells (rho = − 0.240), NK cells (rho = − 0.155), macrophages (rho = − 0.312), eosinophils (rho = − 0.38), monocytes (rho = − 0.318), and neutrophils (rho = − 0.344) (*P* < 0.001). BUB1 was significantly associated with immunomodulators and immunosuppressive agents in EC in TISIDB database online analysis (Fig. 4B). For example, BUB1 was associated with ADORA2A (rho = − 0.232), SLAMF4 (rho = − 0.251), CSF1R (rho = − 0.263), LGALS9 (rho = − 0.298), TGFB1 (rho = − 0.251), and CD160 (rho = − 0.209) (*P* < 0.001). BUB1 was also significantly associated with multiple immunostimulatory factors, including CD27 (rho = − 0.26), CD40LG (rho = − 0.301), HHLA2 (rho = − 0.286), NT5E (rho = − 0.324), TNFRSF14 (rho = − 0.389), and TNFRSF4 (rho = − 0.311) (*P* < 0.001) (Fig. 4C).

BUB1B also correlated strongly with the abundance of multiple TILs (Fig. 4D). BUB1B expression levels correlated with act-CD8 + T cells (rho = − 0.146), act-CD4 + T cells (rho = 0.556), act-B cells (rho = − 0.237), NK cells (rho = − 2.76), macrophages (rho = − 0.347), eosinophils (rho = − 0.381), monocytes (rho = − 0.356), neutrophils (rho = − 0.388), and act-dendritic cells (rho = -0.159) (*P* < 0.001). BUB1B was significantly associated with multiple immunosuppressive factors (Fig. 4E), including ADORA2A (rho = − 0.283), CD244 (rho = − 0.232), CSF1R (rho = − 0.357), PVRL2 (rho = − 0.248), TGFB1 (rho = − 0.272), and LGALS9 (rho = − 0.319) (*P* < 0.001). BUB1B was also significantly associated with multiple immunostimulatory factors, including C10orf54 (rho = − 0.317), RAET1E (rho = − 0.29), TNFRSF14 (rho = − 0.379), TNFSF14 (rho = − 0.377), TNFRSF4 (rho = − 0.318), and TMEM173 (rho = − 0.286) (*P* < 0.001) (Fig. 4F).

### BUB1 and BUBIB affects multiple pathways in tumors

We used the STRING database and obtained 50 BUB1-binding and BUB1B-binding proteins (Fig. S5A,D). The top five genes most strongly associated with BUB1 were NCAPH (r = 0.93), SGOL1 (r = 0.93), DLGAP5 (r = 0.94), CKAP2L (r = 0.94), and KIF11 genes (r = 0.93) (Fig. S5B). The top five genes most strongly associated with BUB1B were NUSAP1(r = 0.93), BUB1 (r = 0.93), OIP5 (r = 0.92), ARHGAP11A (r = 0.92), and KIF11 genes (r = 0.93) (Fig. S5E).

The GO/KEGG enrichment analysis revealed that the main pathways affected by BUB1 and related proteins in tumors were protein homogenization, nuclear chromosome segregation, response to topologically incorrect proteins, response to unfolded proteins, and ER-associated misfolded proteolysis protein catabolic process. The pathways affected by BUB1 and related proteins included protein metabolic processes in tumors (Fig. S5C, Table S2). The pathways mainly affected by BUB1B and related proteins in tumors were cell cycle, DNA replication, microtubule motor activity, microtubule binding, ATPase activity, nuclear division, chromosome segregation, and mitotic nuclear division (Fig. S5F, Table S3). BUB1B and related proteins mainly affect cell cycle proteins in tumors.

### BUB1 and BUBIB expressions are related to clinicopathologic factors

BUB1 and BUB1B expressions were significantly higher in EC in all cancer stages (*P* < 0.01), with higher expression in middle to late stage cancer than early-stage cancer. BUB1 and BUB1B were highly expressed in EC in pre-menopausal, peri-menopausal and post-menopausal stages (*P* < 0.01). BUB1 and BUB1B expressions were significantly higher in serous cases compared with endometrioid tissue cases (*P* < 0.01). BUB1 and BUB1B expressions were higher in TP53-mutated EC compared with TP53 wild-type EC (Table 1).

**Table 1:** The relation between BUB1 and BUB1B expression with clinicopathological characteristics in EC.

Characteristic	n	BUB1 (n, %)		*p*	BUB1B (n, %)		*p*
		Low	High		Low	High	
Total	552	276	276		276	276	
Age, years				0.046			0.447
≤ 60	276	115 (20.9%)	91 (16.6%)		108 (19.7%)	98 (17.9%)	
> 60	343	160 (29.1%)	183 (33.3%)		167 (30.4%)	176 (32.1%)	
Histologic grade				< 0.001			< 0.001
G1	98	80 (14.8%)	18 (3.3%)		73 (13.5%)	25 (4.6%)	
G2	120	85 (15.7%)	35 (6.5%)		77 (14.2%)	43 (7.9%)	
G3	323	107 (19.8%)	216 (39.9%)		123 (22.7%)	200 (37%)	
Clinical stage				< 0.001			0.002
Stage I	342	194 (35.1%)	148 (26.8%)		192 (34.8%)	150 (27.2%)	
Stage II	51	24 (4.3%)	27 (4.9%)		24 (4.3%)	27 (4.9%)	
Stage III	130	47 (8.5%)	83 (15%)		48 (8.7%)	82 (14.9%)	
Stage IV	29	11 (2%)	18 (3.3%)		12 (2.2%)	17 (3.1%)	
Tumor invasion (%)				0.024			0.374
< 50	259	152 (32.1%)	107 (22.6%)		143 (30.2%)	116 (24.5%)	
≥ 50	225	103 (21.7%)	112 (23.6%)		109 (23%)	106 (22.4%)	
Histological type				< 0.001			< 0.001
Endometrioid	410	238 (43.1%)	172 (31.2%)		229 (41.5%)	181 (32.8%)	
Mixed	24	10 (1.8%)	14 (2.5%)		8 (1.4%)	16 (2.9%)	
Serous		28 (5.1%)	90 (16.3%)		39 (7.1%)	79 (14.3%)	

### Knockdown of BUB1 and BUBIB influences proliferation, migration, and invasion of EC cells

**Figure 5:**
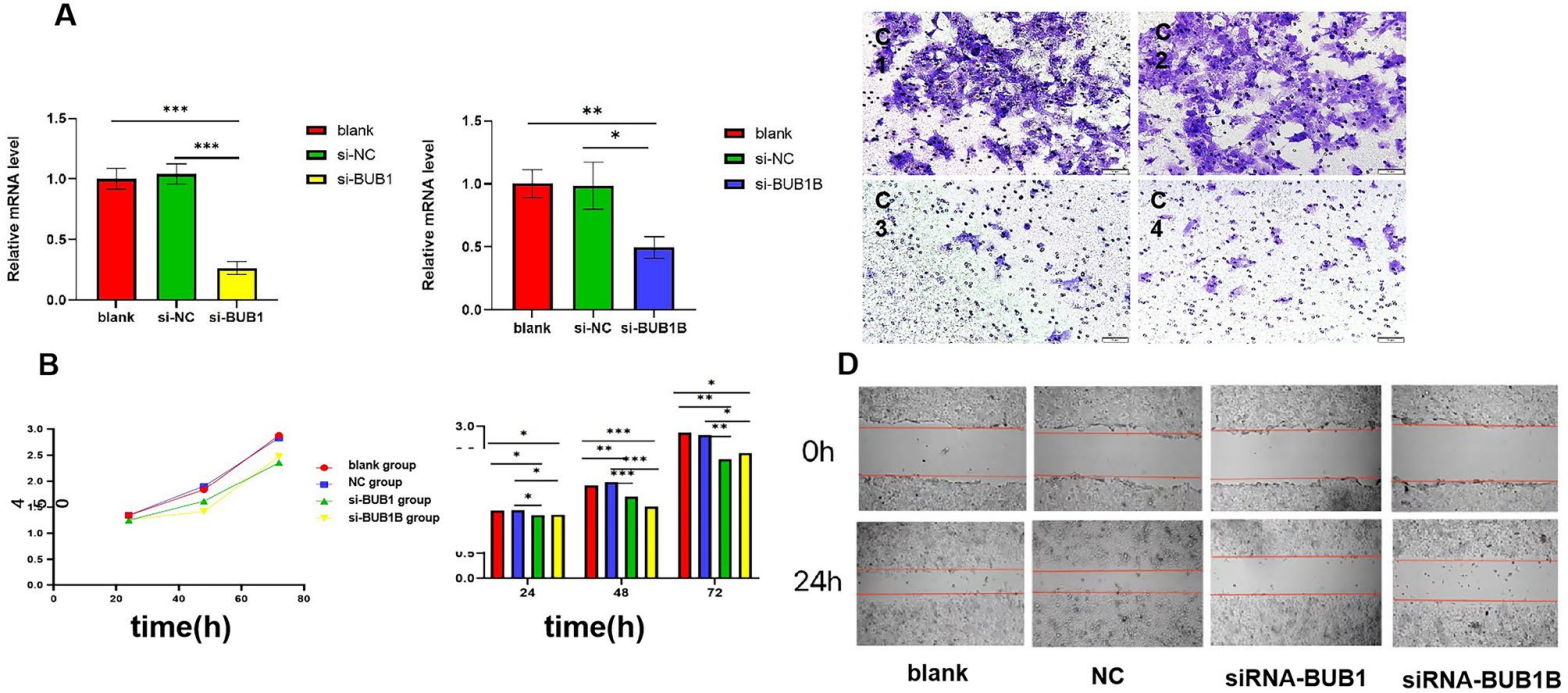
Influence of BUB1 and BUBIB knockdown on EC cell phenotype. (**A**) The growth of Ishikawa cells in each group; (**B**) The migration rates of Ishikawa cells in each group: blank group (50.99 ± 1.02)%, NC-siRNA group (66.50 ± 7.74)%, BUB1-siRNA group (34.87 ± 4.26)%, and BUB1B-siRNA group (31.96 ± 4.72)%; (**C**) The number of cells that crossed the membrane in each group: blank group (127.3 ± 4.8), NC-siRNA group (125.3 ± 3.9), BUB1-siRNA group (48.6 ± 3.2), and BUB1B-siRNA group (35.0 ± 2.1). *P < 0.05, **P < 0.01, ***P < 0.001.

We used siRNA to downregulate BUB1 and BUB1B in EC cells. The relative expression of BUB1 mRNA in the BUB1-siRNA group was significantly lower than the blank group and negative control group (*P* < 0.001), and the relative expression of BUB1B mRNA in the BUB1B-siRNA group was significantly lower than the blank group and NC group (*P* < 0.05), indicating the efficacy of gene knockdown (Fig. 5A).

Cell growth in the siRNA-BUB1 and siRNA-BUB1B groups was significantly inhibited compared with the blank group and NC group (*P* < 0.05) (Fig. 5B). In scratch assays, the wound healing rates of the BUB1-siRNA group (34.87 ± 4.26)% and BUB1B-siRNA group (31.96 ± 4.72)% after 24 h were significantly decreased compared with the blank group (50.99 ± 1.02)% and NC-siRNA group (66.50 ± 7.74)% (*P* < 0.05) (Fig. 5D). In invasion assays, the numbers of invaded cells in the si-BUB1 group and si-BUB1B group were 48.6 ± 3.2 and 35.0 ± 2.1, respectively, which were less than those in the blank group and negative group (127.3 ± 4.8 and 125.3 ± 3.9, respectively), indicating that invasion ability was significantly decreased upon BUB1 and BUB1B knockdown (*P* < 0.05) (Fig. 5C1–4).

## Discussion

EC is one of the most lethal malignancies and shows a poor prognosis because of the high tendency for recurrence and metastasis^24^. Currently, the efficacy of clinical drugs and conventional treatments is limited. Therefore, there is an urgent need for early diagnostic methods and more effective treatments to improve the diagnosis and survival rates of EC patients.

Our results showed that the expressions of BUB1 and BUB1B were significantly upregulated in EC tissues compared with normal endometrial tissues. We further investigated the roles of BUB1 and BUB1B in Ishikawa cells using siRNA and found that silencing BUB1 and BUB1B inhibited cell proliferation, migration and invasion, indicating that BUB1 and BUB1B may exhibit tumor-promoting functions in EC.

Our analyses showed that high BUB1 expression was associated with OS in eight cancers including ACC, KIRC and KIRP and DFS in 10 cancers including ACC and KIRC. High BUB1B expression was associated with OS in nine cancers, including ACC and KIRC and DFS in 11 cancers, including ACC, CHOL and KIRC. These results indicate that high expressions of BUB1 and BUB1B are closely associated with poor prognosis in different malignancies and these proteins may be potential prognostic markers for tumors.

Aneuploidy, a copy number change encompassing an entire chromosome arm or chromosome, is the most common genetic alteration in cancer. Thus, aneuploidy-targeted drugs have been a long sought-after approach in cancer therapy^25^. The expressions of SAC family genes are often dysregulated in cancer cells, leading to chromosomal instability and abnormal chromosome segregation, which results in aneuploidy and promotes tumor development and drug resistance^26–28^. Diminished SAC function contributes to the survival of cancer cells^29,30^. BUB1 and BUB1B are multifaceted kinases involved in the SAC that ensure high fidelity of chromosome segregation in the cell cycle. BUB1 mutation frequently occurs in cancer and leads to tumorigenesis. BUB1B truncation and missense mutations have been found in the mosaic heterozygous aneuploidy family^31,32^. Changes in the expression of BUB1 and BUB1B often lead to impaired SAC function, resulting in deregulation of the cell division cycle and altered cell behavior^33^.

Qin et al.^21^. found six significant hub differentially expressed genes in epithelial ovarian cancer associated with a poor prognosis, including CCNB1, CCNA2, AURKA, BUB1, BUB1B, and CDK1 genes, using bioinformatics analysis. Feng et al.^34^. Reported four genes (BUB1B, BUB1, TTK and CCNB1) that were up-regulated DEGs in ovarian cancer associated with poor prognosis using integrated bioinformatical methods. All three studies (our study and the two cited above) used Kaplan–Meier plotter and Gene Expression Profiling Interactive Analysis for survival analysis of BUB1 and BUB1B and obtained similar results. In our study^22^ we further used an in vitro cell line and clinical specimens to evaluate the expression, clinical significance and functions of BUB1 and BUB1B in endometrial carcinoma.

Approximately 2% of cancer patients have BUB1 gene mutations, including deletions, amplifications and deep deletions. CNVs play key roles in tumor biology and therapeutic response^35^. The most common CNVs in BUB1 were diploidy, gain and mild loss. Approximately 2% of cancer patients have BUB1B gene mutations; the main types were deletion mutations, profound deletions and amplifications. The most common CNVs of BUB1B were diploid, gain-of-function mutations and mild loss. BUB1 and BUB1B mutations have been associated with an increased risk of colon cancer^36,37^ but have not been linked with other cancers. The types and significance of the mutations in different tumors require further exploration.

Comprehensive tumor immune analysis has profound implications for the discovery of effective tumor immunotherapies^38,39^. Immune infiltration of the tumor microenvironment could alter the clinical outcome of malignancies. CAFs are major components of the tumor cell stroma; these cells are associated with different cancer subtypes and help to stratify and tailor therapy^40^. Endothelial and neutrophil cell infiltration has a significant impact on cancer development. BUB1 expression levels were negatively correlated with CAF infiltration in BRCA and TGCT and endothelial cell infiltration in BRCA, KIRC, LUAD, STAD, THCA, and THYM and positively correlated with COAD neutrophil cell infiltration and KIRP and LGG endothelial cell infiltration. BUB1B expression level was negatively correlated with CAF infiltration in BRCA, HNSC-HPV+, TGCT, and THYM and endothelial cell infiltration in BRCA, KIRC, LUAD, STAD, and THYM. Pan-cancer analysis revealed that high expression of BUB1 and BUB1B was associated with poor prognosis and associated with immune infiltration in various cancers.

Numerous trials have demonstrated the safety and efficacy of immunotherapy in EC^41^. Heterogeneous immune infiltration has been observed in EC with differences between tumor grades and molecular subtypes. Natural killer T cells and T cells were significantly associated with the survival of EC patients^42^^.^ Previous studies have found that BUB1 and BUB1B are closely associated with immune infiltration in malignant tumors^43–45^. Our results showed a strong correlation between BUB1 and BUB1B and the abundance of multiple TILs, suggesting that BUB1 and BUB1B play a crucial role in the immune infiltration of EC. Immunomodulators significantly affect the function of immune cells. BUB1 and BUB1B were found to be significantly associated with multiple immunosuppressants and immunostimulants in EC. These findings suggested that BUB1 and BUB1B are closely associated with immunomodulation in EC and may mediate immune escape in tumors.

This study included a small number of cases, which is a limitation of the study. The focus of the immunohistochemical experiments was to investigate expression of BUB1 and BUB1B in endometrial cancer and normal endometrial tissue, and thus only 20 cases were selected. The relationship of BUB1 and BUB1B with clinicopathology and prognosis was subsequently analyzed using bioinformatic approaches and predicted pathway mechanism of them single gene silencing in vitro, but no conducted experimental demonstration. BUB1B is a paralogue gene of BUB1, both of them are important members of SAC family, but whether the two have synergistic effects has not been revealed. Our next step is to collect more cases and explore BUB1 and BUB1B expression in EC in a larger sample set and in different stages.

In summary, we found that BUB and BUB1B influence EC proliferation, migration and invasion and play an important role in immune infiltration. The abnormal expression of BUB1 and BUB1B may play a key role in the development of EC. BUB1 and BUB1B may be biomarkers for the screening, diagnosis and treatment of EC. Further experimental evidence is needed to investigate the specific mechanism of BUB1 and BUB1B in EC.

## Data availability 

The datasets generated during and/or analysed during the current study are available from the corresponding author on reasonable request.

### Supplementary Information

Supplementary Figures.

Supplementary Tables.
