# Supplementary material for: Correlation of BUB1 and BUB1B with the development and prognosis of endometrial cancer
Source: Sci Rep. 2024 Jul 24;14:17084. doi: 10.1038/s41598-024-67528-2 (PMC11269704; doi:10.1038/s41598-024-67528-2)
Supplement: Supplementary file 1 — Supplementary Figures. [file 41598_2024_67528_MOESM1_ESM.docx]

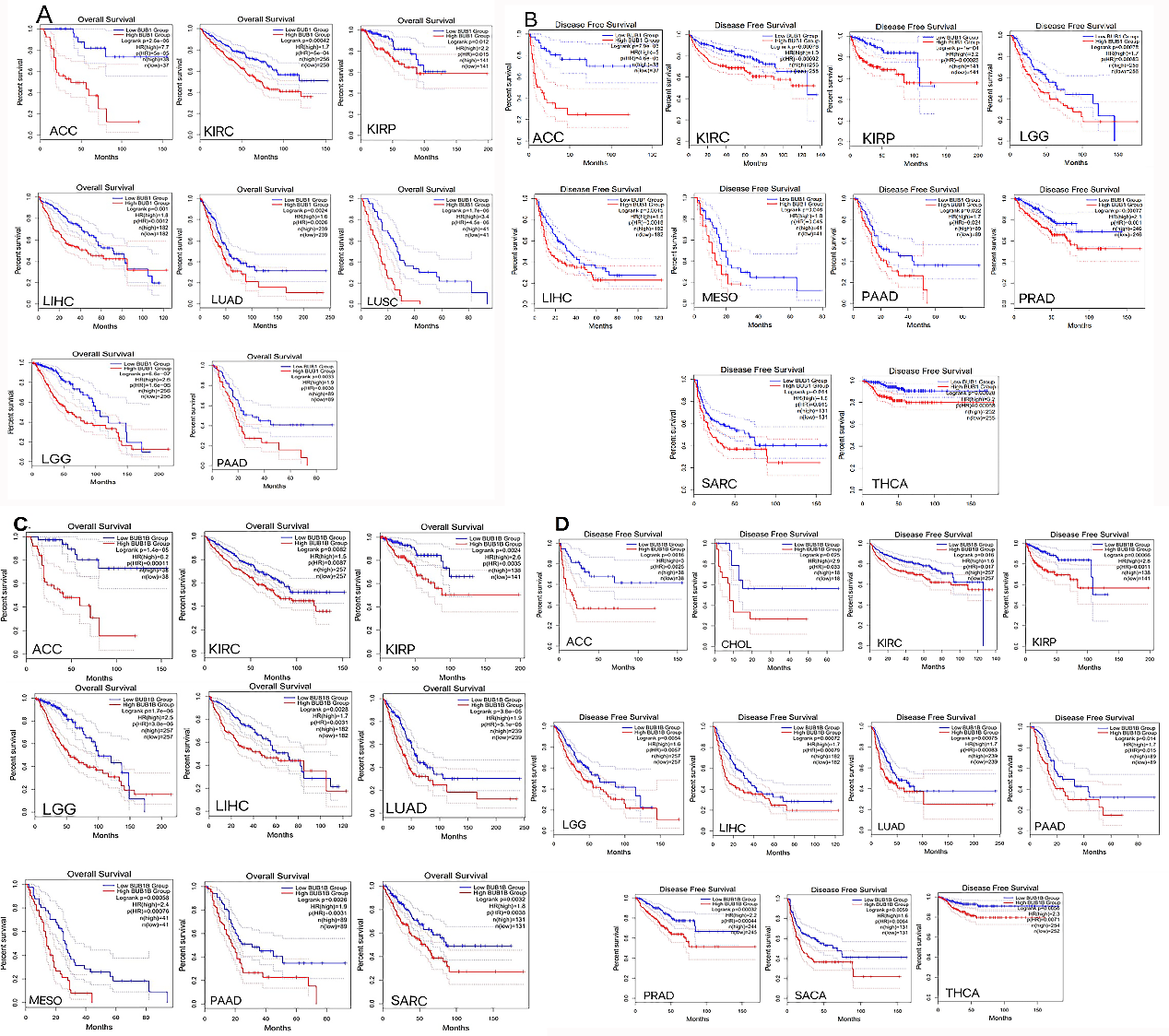
Figure S1. Relationship between BUB1 and BUB1B levels with prognosis in different cancers.

A. Relationship between BUB1 level and OS in different cancers; B. Relationship between BUB1 level and DFS in different cancers. C. Relationship between BUB1B level and OS in different cancers; D. Relationship between BUB1B level and DFS in different cancers.


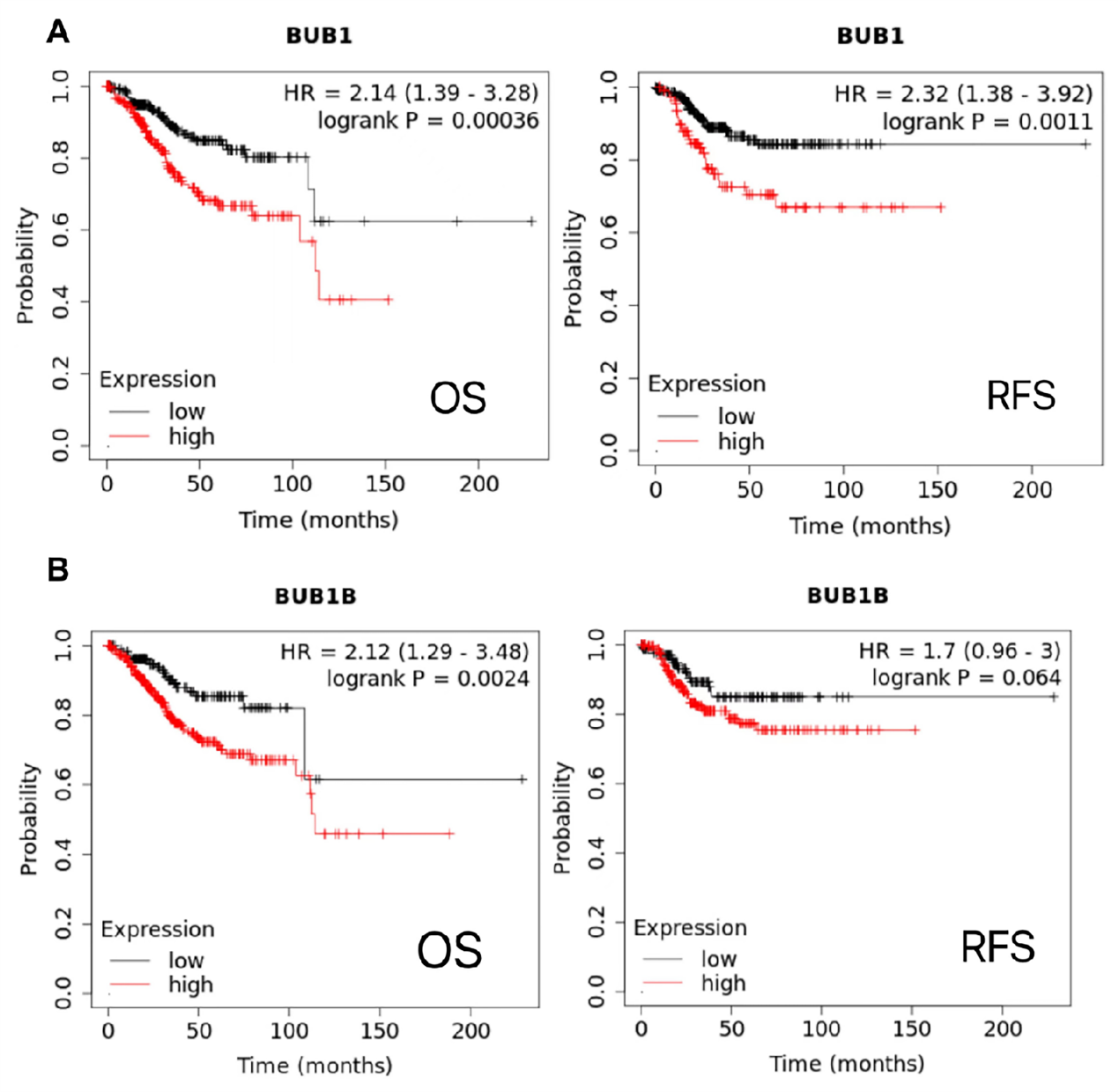


Figure S2. BUB1 or BUB1B mRNA level and prognosis of EC

A. BUB1 mRNA level and prognosis of EC; B. BUB1B mRNA level and prognosis of EC.


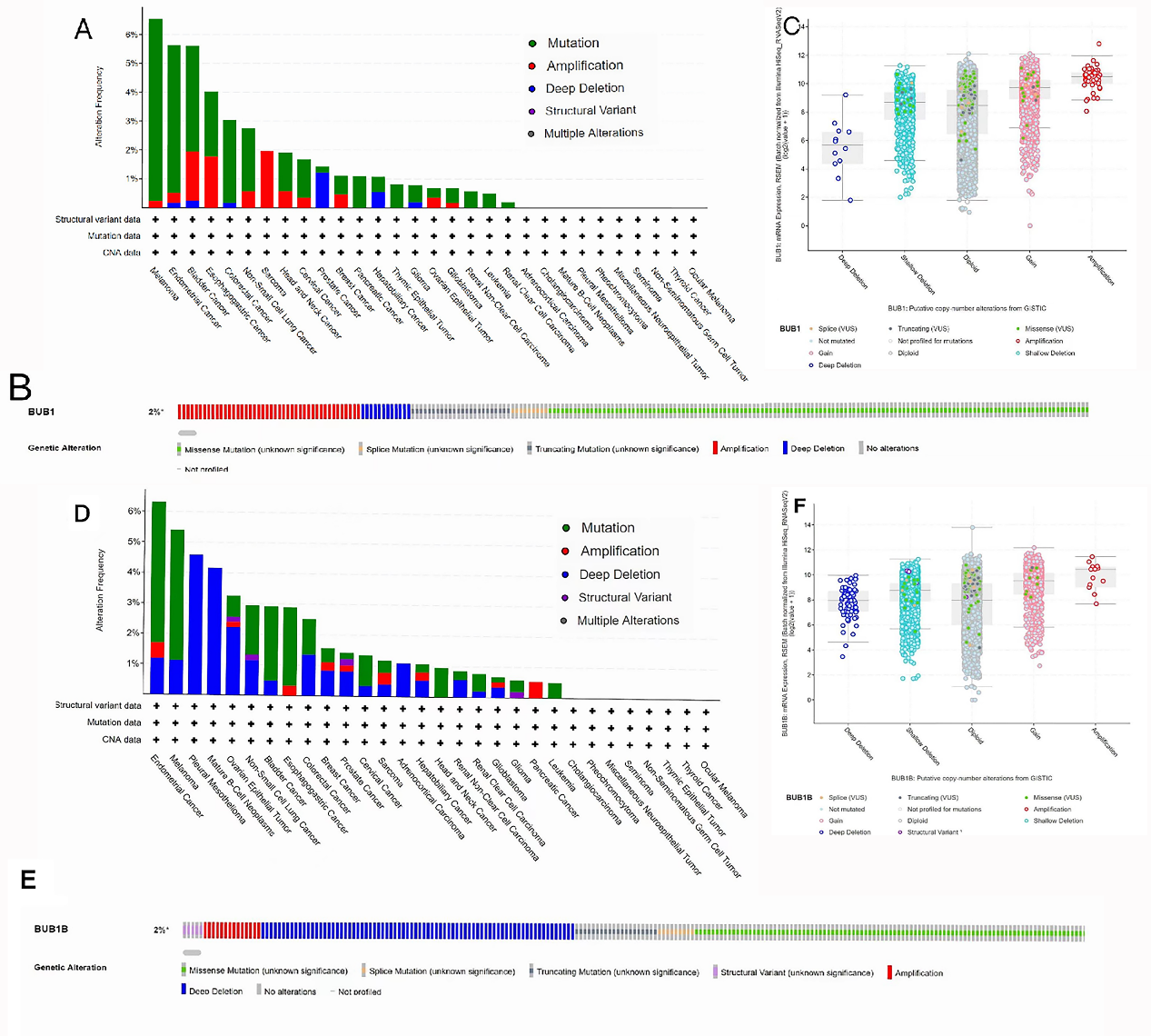


Figure S3. Genetic variations of BUB1 and BUB1B genes in tumors.

A. BUB1 gene alteration in different tumors; B. The main types of BUB1 genetic variations; C. The main CNV types of BUB1 gene; D. BUB1B gene alteration in different tumors; E. The main types of BUB1B genetic variations; F. The main CNV types of BUB1B gene.


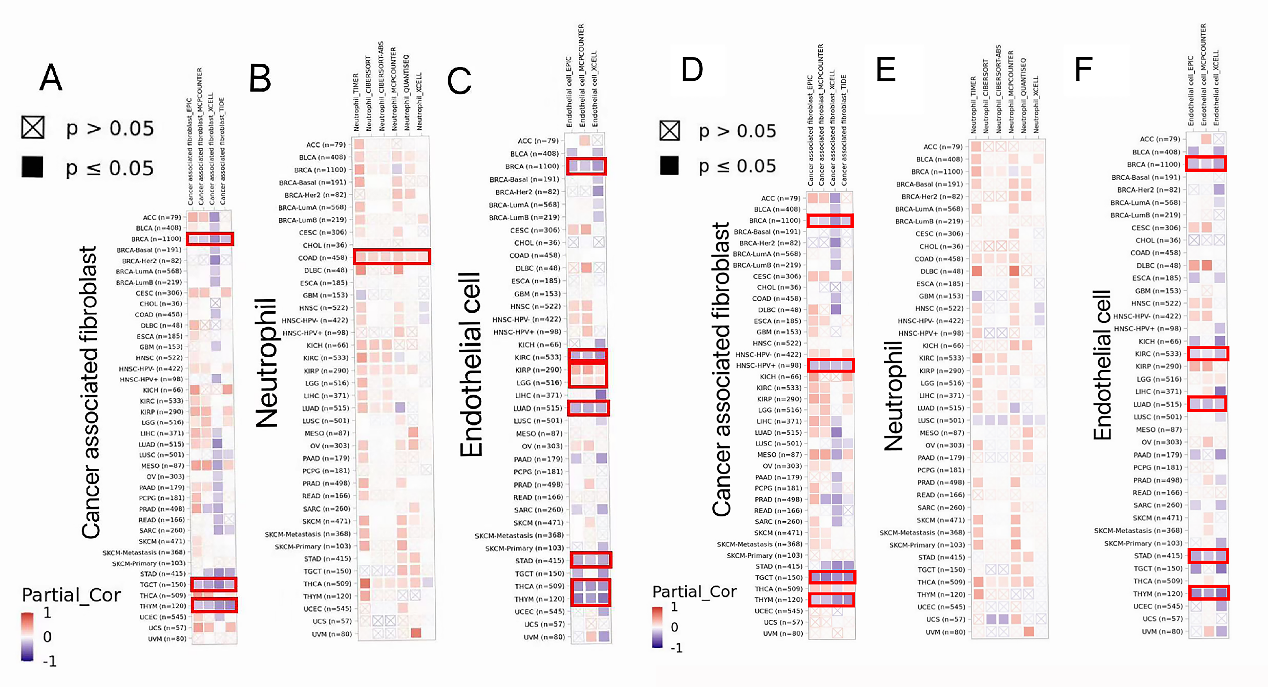
Figure S4. BUB1 enrichment analysis

A. BUB1 binding protein and protein network diagram (STRING database); B. Pearson analysis of BUB1 and the five genes with the strongest correlation in various tumors (GEPIA2 database); C. GO/KEGG analysis based on BUB1 binding and interacting genes in various tumors (XiaoTao database).


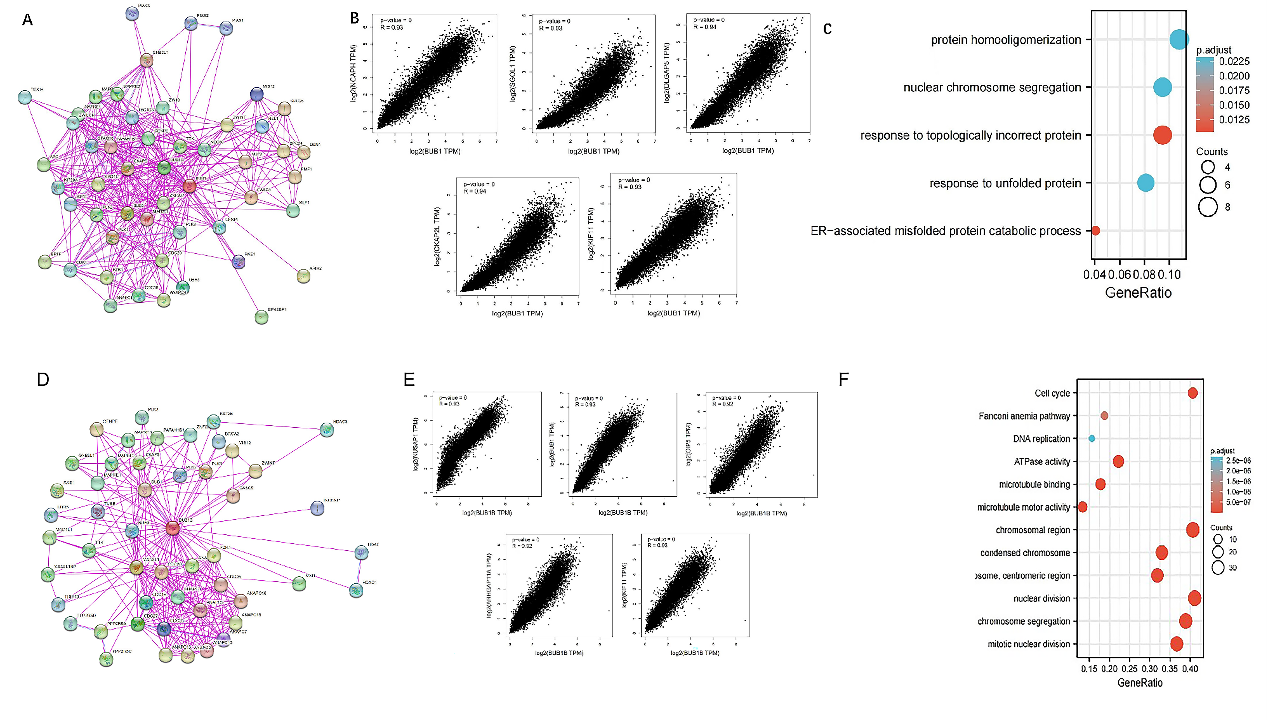


Figure S5. BUB1B enrichment analysis

A. BUB1B binding protein and protein network diagram (STRING database); B. Pearson analysis of BUB1B and the five genes with the strongest correlation in various tumors (GEPIA2 database); C. GO/KEGG analysis based on BUB1B binding and interacting genes in various tumors (XiaoTao database).
