# Supplementary material for: Correlation of BUB1 and BUB1B with the development and prognosis of endometrial cancer
Source: Sci Rep. 2024 Jul 24;14:17084. doi: 10.1038/s41598-024-67528-2 (PMC11269704; doi:10.1038/s41598-024-67528-2)
Supplement: Supplementary file 2 — Supplementary Tables. [file 41598_2024_67528_MOESM2_ESM.docx]

Table S1. Primer sequences

|  | Forward | Reverse |
| --- | --- | --- |
| BUB1 | GAAGAAATACCACAATGACCCAAG | TGGGTTTCAGTCAGGCGTGT |
| BUB1B | TGCTTCCCAGTTTCACTCCATA | CCTTCTTCTTTCCAGGCTTTC |
| GADPH | GGAAGCTTGTCATCAATGGAAATC | TCATGACCCTTTTGGCTCCC |

Table S2. BUB1 co-expression and the interacting protein network of GO biological process analysis

| Term-ID | Biological process | False rate |
| --- | --- | --- |
| GO:1903033 | Positive regulation of microtubule plus-end binding | 0.0030 |
| GO:1905786 | Positive regulation of anaphase-promoting complex-dependent catabolic process | 0.0047 |
| GO:1904825 | Protein localization to microtubule plus-end | 0.00010 |
| GO:0007094 | Mitotic spindle assembly checkpoint | 1.05e-20 |
| GO:0051745 | Meiotic sister chromatid cohesion | 0.0067 |
| GO:0051315 | Attachment of mitotic spindle microtubules to kinetochore | 6.84e-08 |
| GO:0008608 | Attachment of spindle microtubules to kinetochore | 9.26e-15 |
| GO:0034501 | Protein localization to kinetochore | 1.62e-09 |
| GO:0016321 | Female meiosis chromosome segregation | 0.0090 |
| GO:0090166 | Golgi disassembly | 0.0118 |

Table S3. BUB1B co-expression and GO biological process analysis of the interacting protein network

| Term-ID | Biological process | False rate |
| --- | --- | --- |
| GO:1903033 | Positive regulation of microtubule plus-end binding | 0.0024 |
| GO:0007091 | Metaphase/anaphase transition of mitotic cell cycle | 4.90e-11 |
| GO:1905786 | Positive regulation of anaphase-promoting complex-dependent catabolic process | 0.0038 |
| GO:0061198 | Fungiform papilla formation | 0.0038 |
| GO:1904825 | Protein localization to microtubule plus-end | 7.31e-05 |
| GO:0051754 | Meiotic sister chromatid cohesion | 0.0054 |
| GO:0045842 | Positive regulation of mitotic metaphase/anaphase transition | 7.95e-10 |
| GO:0007094 | Mitotic spindle assembly checkpoint | 0.0054 |
| GO:0070979 | Protein k11-linked ubiquitination | 7.95e-10 |
| GO:0060789 | Hair follicle placode formation | 1.68e-16 |
